# Assessing Diabetes Self-Management with the Diabetes Self-Management Questionnaire (DSMQ) Can Help Analyse Behavioural Problems Related to Reduced Glycaemic Control

**DOI:** 10.1371/journal.pone.0150774

**Published:** 2016-03-03

**Authors:** Andreas Schmitt, André Reimer, Norbert Hermanns, Jörg Huber, Dominic Ehrmann, Sabine Schall, Bernhard Kulzer

**Affiliations:** 1 Research Institute of the Diabetes Academy Mergentheim (FIDAM), Theodor-Klotzbücher-Str. 12, 97980 Bad Mergentheim, Germany; 2 German Center for Diabetes Research (DZD), 85764 München-Neuherberg, Germany; 3 Otto-Friedrich-University of Bamberg, Department for Psychology, Markusplatz 3, 96047 Bamberg, Germany; 4 Centre for Health Research, University of Brighton, Falmer, BN1 9PH, United Kingdom; Massachusetts General Hospital, UNITED STATES

## Abstract

**Aim:**

To appraise the Diabetes Self-Management Questionnaire (DSMQ)’s measurement of diabetes self-management as a statistical predictor of glycaemic control relative to the widely used SDSCA.

**Methods:**

248 patients with type 1 diabetes and 182 patients with type 2 diabetes were cross-sectionally assessed using the two self-report measures of diabetes self-management DSMQ and SDSCA; the scales were used as competing predictors of HbA_1c_. We developed a structural equation model of self-management as measured by the DSMQ and analysed the amount of variation explained in HbA_1c_; an analogue model was developed for the SDSCA.

**Results:**

The structural equation models of self-management and glycaemic control showed very good fit to the data. The DSMQ’s measurement of self-management showed associations with HbA_1c_ of –0.53 for type 1 and –0.46 for type 2 diabetes (both *P* < 0.001), explaining 21% and 28% of variation in glycaemic control, respectively. The SDSCA’s measurement showed associations with HbA_1c_ of –0.14 (*P* = 0.030) for type 1 and –0.31 (*P* = 0.003) for type 2 diabetes, explaining 2% and 10% of glycaemic variation. Predictive power for glycaemic control was significantly higher for the DSMQ (*P* < 0.001).

**Conclusions:**

This study supports the DSMQ as the preferred tool when analysing self-reported behavioural problems related to reduced glycaemic control. The scale may be useful for clinical assessments of patients with suboptimal diabetes outcomes or research on factors affecting associations between self-management behaviours and glycaemic control.

## Introduction

When it comes to controlling blood glucose levels and establishing euglycaemia in diabetes, the probably most important factor is patients’ self-management of the condition [1–3]. Since poor glycaemic control contributes greatly to the risk of developing serious long-term complications of diabetes [4–7], the improvement of relevant self-management behaviours can be an important key to improved diabetes prognosis and reduced risks of long-term complications.

For clinicians seeking to assess their patients’ diabetes self-management and detect potential areas in need of improvement, a standardised psychometric assessment tool which provides reliable and valid assessments of the essential domains of diabetes self-management can be a valuable tool. For researchers who need to measure diabetes self-management as part of a study, a standardised tool may be key. It is therefore not surprising that over the last three decades a variety of self-report measures concerning diabetes self-management have been developed. A recent systematic review identified a total of 21 multidimensional scales of overall diabetes self-management plus some additional ones focussing on single self-management domains, originating from various countries [8]. However, despite this seeming abundance of measurement options, the number of useful and psychometrically satisfactory instruments is indeed limited. Moreover, not all of the tools reviewed in the paper are at all accessible. Out of the 21 scales, only ten were available in English language, and out of those, only five were rated sufficiently validated by the reviewing authors [8]. This result clearly corresponds to two earlier reviews on the topic which collected a total of five [9] and seven [10] self-management scales, respectively.

One of the few instruments to satisfy comprehensive psychometric appraisal is the Summary of Diabetes Self-Care Activities Measure (SDSCA). It is in fact the only scale judged positively by all three reviews, and although not free from weaknesses [8,10], it is the only tool which met the rigorous appraisal criteria of the review by Eigenmann et al. [9]. Moreover, the SDSCA has gained the status of a standard measure, similar to a gold standard, since its development in 1993 and revision in 2000 [11,12], as it is the most commonly used research tool to assess diabetes self-management today [9,13]. Its efficiency and economy with no more than eleven items to cover five essential self-management domains–general diet, specific diet, exercise, blood-glucose testing and foot care–has proven to be appealing to both researchers and clinicians.

However, the SDSCA has several limitations, the most important one being a poor association with levels of glycaemic control. In fact, an absence of significant associations between self-management behaviours as measured by the SDSCA and HbA_1c_ was already reported in the scale’s initial evaluation [11]; subsequent studies supported this result with findings of generally low associations between the SDSCA and HbA_1c_ [13–18]. This limitation is problematic for two reasons: 1) Since we assume that better self-management (e. g. adjusting diet; testing blood glucose; taking required medications) should lead to better glycaemic control, a weak association between these variables raises concerns regarding the measure’s validity. 2) Such a weak association limits the measure’s practical utility for both clinicians and researchers. For example, a number of studies aimed to analyse the impact of psychosocial factors such as depression, which is supposed to cause reduced glycaemic control through disregard of diabetes self-management, on these outcomes in comprehensive regression or structural equation model analyses [17–20]. However, studies using the SDSCA were usually inconclusive [17–20], which appears comprehensible when keeping the weak associations with HbA_1c_ in mind. When seeking to understand self-reported behavioural causes of hyperglycaemia in a patient or when aiming to analyse aspects of self-management and glycaemic control in studies, a measurement tool with good predictive power of glycaemic outcomes is to be preferred.

A relatively new psychometric tool to assess diabetes self-management is the Diabetes Self-Management Questionnaire (DSMQ), introduced in 2013 [21]. The scale covers several important domains–diet, medication, blood glucose monitoring, physical activity and contact with health-care professionals–and particularly focusses on activities related to glycaemic control. It was thoroughly evaluated and has since then been used in studies in Germany [22–24], the UK [25,26] and the US [27–29], i. a. However, it was not included in any of the aforesaid reviews due to its relative novelty. There is evidence of above-average convergence between the self-management behaviours measured by the DSMQ and glycaemic control. This suggests good eligibility and utility of this tool either for analysing behavioural problems related to hyperglycaemia in clinical practice or for testing putative mediation of the effect of a given factor on glycaemic control by diabetes self-management.

Following this evidence, we planned to rigorously test the DSMQ as a statistical predictor of glycaemic control in patients with type 1 and type 2 diabetes using structural equation modelling. We hypothesized to find significantly greater predictive power (i. e. linear association with HbA_1c_ and explanation of glycaemic variation, respectively) by diabetes self-management as measured using the DSMQ compared to self-management as measured using the common standard, SDSCA.

## Material and Methods

Data acquisition was performed as part of a larger study, approved by the Ethics Committee of the State Medical Chamber of Baden-Wuerttemberg. A cross-sectional convenience sample of 430 patients with type 1 or type 2 diabetes was enrolled at a referral centre for people with diabetes in Germany (Diabetes Center Mergentheim). Inclusion criteria were adult age (≥ 18 years), diabetes type 1 or type 2, sufficient language skills and written informed consent. Exclusion criteria were terminal illness, being bedbound and being under guardianship (based on patient records). Patients eligible for study participation were approached during their 10 to 12 days stay at the centre, informed about the study and invited to participate. The participation rate amounted to 62%. Study participants were assessed using the DSMQ and SDSCA. HbA_1c_ was analysed in the centre’s laboratory simultaneously with the questionnaire assessments. Demographic data were collected in interviews conducted by the centre’s nurses. Long-term complications were diagnosed by the centre’s physicians.

### Instruments and Measures

#### Diabetes Self-Management Questionnaire (DSMQ)

The DSMQ consists of 16 items covering five different aspects of diabetes self-management. All items are formulated as behavioural descriptions from the person’s point of view (examples below). Respondents rate the extent to which each description applies to them on a four-point Likert scale (3 –‘applies to me very much’ to 0 –‘does not apply to me’), referring to the previous eight weeks (full contents are provided in S1 Table). Item scores are transformed so that higher scores indicate more desirable self-management behaviour (requiring reverse-scoring of negatively-keyed items) and summed/transformed to five scale scores with ranges from 0 to 10 (scoring is explained in detail elsewhere [21]). The scales reflect patients’ *dietary control* (4 items; e.g. ‘The food I choose to eat makes it easy to achieve optimal blood sugar levels’), *medication adherence* (2 items; e.g. ‘I tend to forget or skip my diabetes medication’), *blood glucose monitoring* (3 items; e.g. ‘I check my blood sugar levels with care and attention’), *physical activity* (3 items; e.g. ‘I am less physically active than would be optimal for my diabetes’) and *physician contact* (3 items; e.g. ‘I keep all doctors’ appointments recommended for my diabetes treatment’). Its validation study supported the DSMQ’s reliability and validity [21]; in the present study, reliability coefficients were observed as follows (Cronbach’s *α*; stratified by scale): dietary adherence 0.79; medication adherence 0.75; blood glucose monitoring 0.83; physical activity 0.74; appointment adherence 0.72.

#### Summary of Diabetes Self-Care Activities Measure (SDSCA)

The SDSCA is a standard self-report scale to assess diabetes self-management. Ten items assess the frequencies of specific self-management activities during the previous week; an additional item assesses smoking. Respondents mark the numbers of days (0–7) on which the indicated behaviours were performed. The item scores can be averaged to the five subscales *general diet* (2 items; e.g. ‘How many of the last seven days have you followed a healthful eating plan?’), *specific diet* (2 items; e.g. ‘On how many of the last seven days did you eat five or more servings of fruits and vegetables?’), *exercise* (2 items; e.g. ‘On how many of the last seven days did you participate in at least 30 minutes of physical activity?’), *blood-glucose testing* (2 items; e.g. ‘On how many of the last seven days did you test your blood sugar?’) and *foot care* (2 items; e.g. ‘On how many of the last seven days did you check your feet?’). All scale scores range from 0 to 7 with higher scores suggesting better self-management. The SDSCA has shown adequate reliability and validity in English [12] as well as German samples [13]; in this study, reliability coefficients were observed as follows (Cronbach’s *α*; stratified by scale): general diet 0.89, specific diet 0.30, exercise 0.74, blood-glucose testing 0.78, foot care 0.72.

#### Commonalities and differences of the DSMQ and the SDSCA

When comparing the DSMQ and SDSCA, there is significant overlap in contents with regard to the aspects diet, exercise/activity and self-monitoring of blood glucose. Nevertheless, the measurement approach is different since the SDSCA requests numbers of days per last week on which a particular behaviour was performed. Hence, the appraisal of good versus poor self-management lies in the hands of the professional and must be based on the patient’s individual regimen. By contrast, the DSMQ involves the self-rating of self-management by the patient, and rating is done with regard to a more general time period of eight weeks. Results may be more representative but potentially also more susceptible to memory bias. Furthermore, the DSMQ comprises items on medication intake and contact to one’s physician–aspects which were considered relevant regarding glycaemic control when developing the scale. In its earlier version, the SDSCA also included an item on medication, but this was excluded due to psychometric limitations when the scale was revised to its current form in 2000 [12]. On the other hand, the SDSCA includes foot care and smoking behaviours, which represents a relevant difference to the DSMQ. In sum, the two scales differ significantly regarding assessed timeframe, item formulation and item content.

#### Glycated haemoglobin

As a measure of patients’ glycaemic control, glycated haemoglobin (HbA_1c_) was assessed. This measure reflects a patient’s average blood glucose levels during the previous two to three months [30]. All blood samples were analysed in a central laboratory at the same time point as the questionnaire assessment were conducted (i. e. assessments were made within one to seven days apart from each other, on average about three days). HbA_1c_ was determined using high performance liquid chromatography (HPLC) performed with the Bio-Rad Variant II Turbo analyser (meeting the requirements of current standards of HbA_1c_ measurements; DCCT standard). The laboratory normal range is 4.3–6.1% (23.5–43.2 mmol/mol).

### Statistical Analyses

**Fig 1:**
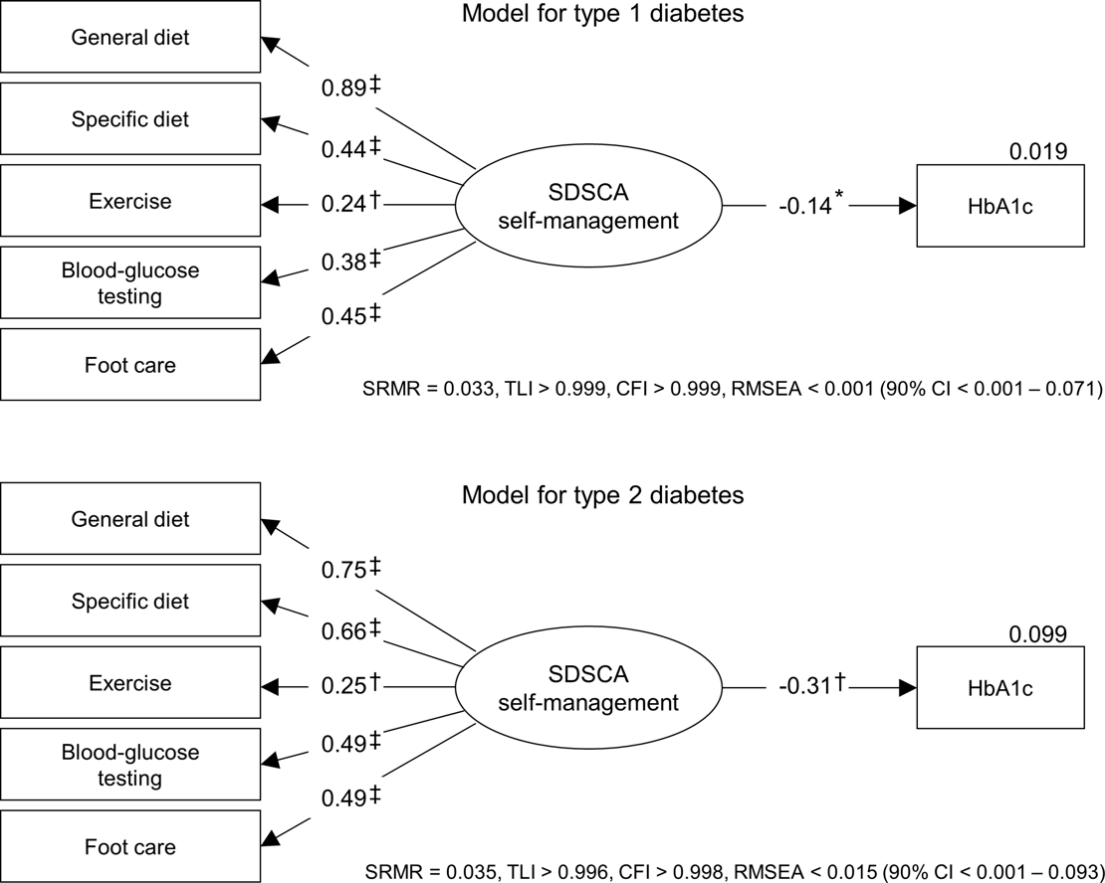
Stuctural equation model of diabetes self-management as measured by the DSMQ and glycaemic control for patients with type 1 and type 2 diabetes. Data are standardised regression weights (*β*) for paths or squared multiple correlations (*R*^2^) for variables. Boxes indicate manifest measurement variables; ovals indicate latent variables operationalised by manifest indicators; error terms are not displayed for ease of presentation. SRMR, Standardised Root Mean Square Residual; TLI, Tucker Lewis Index; CFI, Comparative Fit Index; RMSEA, Root Mean Square Error of Approximation. Indication of two-sided significance: * *P* < 0.05; † *P* < 0.01; ‡ *P* < 0.001.

**Fig 2:**
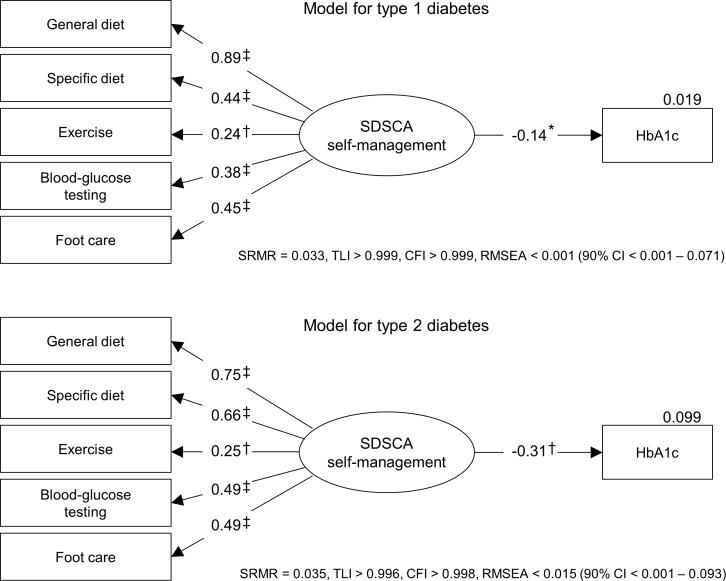
Stuctural equation model of diabetes self-management as measured by the SDSCA and glycaemic control for patients with type 1 and type 2 diabetes. Data are standardised regression weights (*β*) for paths or squared multiple correlations (*R*^2^) for variables. Boxes indicate manifest measurement variables; ovals indicate latent variables operationalised by manifest indicators; error terms are not displayed for ease of presentation. SRMR, Standardised Root Mean Square Residual; TLI, Tucker Lewis Index; CFI, Comparative Fit Index; RMSEA, Root Mean Square Error of Approximation. Indication of two-sided significance: * *P* < 0.05; † *P* < 0.01; ‡ *P* < 0.001.

The statistical analyses were performed using SPSS 22.0.0 including AMOS 22.0.0 (IBM SPSS Statistics, New York, USA). A *P* value < 0.05 was considered as criterion of statistical significance in all analyses. Structural equation modelling was performed using maximum likelihood estimation. The basic test model included the variables HbA_1c_ and diabetes self-management with the latter being modelled as latent variable operationalised by either the DSMQ’s or SDSCA’s five self-management behaviours (see Figs 1 and 2). This approach enabled the integration of the heterogeneous behaviours in a single variable of diabetes self-management, while the individual behaviours were weighted based on the fit to the data. As a result, the structural equation modelling warranted optimal predictive power of diabetes self-management as operationalised by the DSMQ or SDSCA behaviours regarding glycaemic control.

The resulting models were tested on people with type 1 and type 2 diabetes separately, each being revised by successively modelling correlations between the variables’ error terms, following significant modification indices (threshold = 4.0) in order to attain adequate fit of the models. Model fit was evaluated according to the recommendations by Hu and Bentler [31]: Standardised Root Mean Square Residual (SRMR) ≤ 0.08, Tucker Lewis Index (TLI) ≥ 0.95, Comparative Fit Index (CFI) ≥ 0.95 and Root Mean Square Error of Approximation (RMSEA) ≤ 0.06 (with upper bound of the 90% CI ≤ 0.08). The fitted models were used to evaluate the predictive power of the DSMQ and SDSCA regarding HbA_1c_ (assessed by the explanation of variance, i. e. squared multiple correlation, of HbA_1c_). Differences between standardised regression coefficients were tested for statistical significance using Steiger’s *z*-test of the difference between dependent correlations. To account for potential confounding effects, the models were additionally analysed while adjusting for gender and age.

Evaluations of the self-report data’s distribution characteristics revealed non-normal distributions in three of the DSMQ scales (medication adherence, blood glucose monitoring and appointment adherence) and three of the SDSCA scales (exercise, blood-glucose testing and foot care). To ensure adequate normality, all scale scores were converted using Templeton’s two-step method for transforming continuous variables to normal [32]. The procedure involves a first step of percentile rank transformation, resulting in uniformly distributed probabilities, followed by a second step applying the inverse-normal transformation in order to form variables consisting of normally distributed *z*-scores.

## Results

### Sample description

The sample characteristics are displayed in Table 1. The total sample comprised 248 patients with type 1 diabetes (58%) and 182 patients with type 2 diabetes (42%).

**Table 1:** Characteristics of the study sample.

	Total sample	Type 1 DM	Type 2 DM
	*n* = 430 (100%)	*n* = 248 (57.7%)	*n* = 182 (42.3%)
*Demographic variables*:			
Female gender	233 (54.2%)	149 (60.1%)	84 (46.2%)
Age (years)	46.6 ± 14.6	39.0 ± 13.2	57.0 ± 8.9
BMI (kg/m^2^)	30.0 ± 10.0	26.9 ± 11.0	34.1 ± 6.6
*Diabetes-related variables*:			
Diabetes duration (years)	15.3 ± 10.5	16.6 ± 12.1	13.5 ± 7.5
Diabetes treatment:			
Insulin treatment	393 (91.4%)	248 (100%)	146 (80.2%)
Other medical treatment^a^	37 (8.6%)	0 (0%)	36 (19.8%)
HbA_1c_:			
Value in %	8.5 ± 1.6	8.5 ± 1.6	8.6 ± 1.5
Value in mmol/mol	70 ± 17	70 ± 18	70 ± 17
Patients with values > 7.5% (59.5mmol/mol)	308 (71.6%)	176 (71.0%)	132 (72.5%)
Long-term complications:			
Retinopathy	100 (23.3%)	57 (23.0%)	43 (23.6%)
Neuropathy	123 (28.6%)	36 (14.5%)	87 (47.8%)
Nephropathy	44 (10.2%)	11 (4.4%)	33 (18.1%)
Foot ulcer	23 (5.3%)	5 (2.0%)	18 (9.9%)

Data are *n* (%) or M ± SD. BMI, Body Mass Index; HbA_1c_, glycated haemoglobin; M, mean; SD, standard deviation.

^a^ oral antidiabetic agents and/or incretin mimetics.

Patients with type 1 diabetes had a mean (± SD) age of 39 (± 13) years; the mean BMI was 27 (± 11) kg/m^2^, and with a prevalence of 60%, females were slightly overrepresented. Glycaemic control was clearly improvable as 71% of the patients had HbA_1c_ values above 7.5% (59.5 mmol/mol) (the criterion for adequate glycaemic control suggested by the German Diabetes Association). 31% were diagnosed with one or more long-term complications.

Patients with type 2 diabetes were on average 57 (± 9) years old and had a mean BMI of 34 (± 7) kg/m^2^. With a rate of 46% of the sample being female, the gender distribution was relatively balanced. The majority of patients (80%) were treated with insulin, corresponding to the rather long illness duration of 14 (± 8) years. Glycaemic control was comparable to that of the type 1 diabetes group; approximately 73% had HbA_1c_ values above 7.5% (59.5 mmol/mol). Long-term complications were present in 62% of the patients.

### Structural equation model of diabetes self-management as measured by the DSMQ

The structural equation model of diabetes self-management as measured by the DSMQ is displayed in Fig 1. The model showed very good fit for both type 1 and type 2 diabetes (see Fig 1), and all regression weights were significant with *P* < 0.001. Diabetes self-management as operationalised by the DSMQ’s behaviour scales showed a notable negative association with HbA_1c_ amounting to –0.53 (*P* < 0.001) for people with type 1 diabetes and –0.46 (*P* < 0.001) for people with type 2 diabetes. The squared multiple correlations for these associations were 0.276 and 0.213, respectively, equalling an explanation of 27.6% of glycaemic variation for patients with type 1 and 21.3% of variation for those with type 2 diabetes. Most relevant DSMQ behaviours with regard to glycaemic control were blood glucose monitoring, medication adherence and dietary control for both diabetes types, whereas physician contact and physical activity appeared less relevant in this regard (see Fig 1). Adjusting the model for potential confounding effects of demographic variables did not appreciably alter these results.

### Structural equation model of diabetes self-management as measured by the SDSCA

The structural equation model of diabetes self-management as measured by the SDSCA is displayed in Fig 2. The model showed very good fit for both diabetes types (see Fig 2), and all regression weights were significant with *P* < 0.01. Diabetes self-management as operationalised by the SDSCA’s behaviour scales showed a significant negative association with HbA_1c_ of –0.14 (*P* = 0.030) for type 1 diabetes and –0.31 (*P* = 0.003) for type 2 diabetes. The squared multiple correlations between self-management and HbA_1c_ were 0.019 and 0.099, respectively, equalling an explanation of roughly 2% of glycaemic variation for type 1 diabetes and 10% of variation for type 2 diabetes. Most relevant SDSCA behaviours with regard to glycaemic control were general and specific diet as well as blood glucose testing. As was the case with the corresponding DSMQ scale, exercise was less relevant regarding the glycaemic outcome (see Fig 2). Adjusting the model for potential confounding effects of demographic variables did not appreciably alter these results.

### Testing the differences between associations for statistical significance

Steiger’s *z*-test was used to test if the differences between the associations of diabetes self-management as measured by the DSMQ versus SDSCA with HbA_1c_ were statistically significant. The comparison yielded *z*-scores of 10.10 for type 1 and 3.39 for type 2 diabetes, indicating significantly stronger associations between the DSMQ self-management scores and HbA_1c_ compared to those of the SDSCA for both type 1 and 2 diabetes (both *P* < 0.001).

## Discussion

Testing the DSMQ as a statistical predictor of the glycaemic control of patients with type 1 and type 2 diabetes yielded a number of notable findings: First, diabetes self-management as measured by the DSMQ’s self-management behaviours was indeed strongly related to glycaemic control as reflected by HbA_1c_, in total explaining between 21 and 28 percent of glycaemic variation. This finding suggests that the DSMQ may be a valuable assessment tool for professionals who seek to understand causes of hyperglycaemia in patients by evaluating their self-management behaviours. Second, the associations between self-management as measured by the DSMQ were significantly stronger than the equivalent ones for the SDSCA, and the amount of glycaemic variation explained by the DSMQ’s measurement was in fact about twice that of the SDSCA. However, since not all aspects assessed by the latter scale can be assumed to predict glycaemia (e. g. foot care, smoking), this result does not necessarily have to devalue the widely used SDSCA. Nevertheless, it constitutes evidence that when focussing self-management in relation to glycaemic control, the DSMQ should be the measurement of choice. Third, using structural equation modelling for our analyses enabled the integration of heterogeneous self-management behaviours into a single variable of self-management, while weighting the behaviours with regard to their relevance for glycaemic control. Hence, we were able to analyse which behaviours of the DSMQ and SDSCA had a greater impact on the glycaemic outcome. While self-management based on the SDSCA had its highest beta coefficients in dietary behaviours, while other aspects appeared less relevant, self-management as based on the DSMQ showed strong associations not only regarding diet but also blood glucose monitoring and medication adherence. Since these behaviours can be expected to impact on the glycaemic control of people with both type 1 and type 2 diabetes, these differences between the two scales may account for their unequal predictive power regarding HbA_1c_.

Our results also support the DSMQ as a measurement tool of choice in studies aiming to analyse potential impact of specific factors of interest (e. g. depression) on diabetes self-management and glycaemic control using structural equation modelling, as often intended [17–20]. Good model fit, valid operationalisation of self-management by the DSMQ’s behaviour scales and good convergence with HbA_1c_ suggest that a structural equation model analysis of the impact of depression on glycaemic control via self-management, as conducted for example by Lustman et al. [17], might potentially produce more conclusive results when using the DSMQ compared to the SDSCA.

Without a doubt, there are several alternative measures besides these two scales. Some of those have shown promising properties supporting their usability [8–10]. However, overall bivariate correlations between the DSMQ and HbA_1c_ with values between –0.40 and –0.43 [21,22] appear to lie above the average of such correlations observed for most analogous instruments. Studies reporting on this aspect usually found associations of small to moderate sizes [10], specifically between –0.10 and –0.26 for the SDSCA [21,13], between –0.16 and –0.37 for the Self-Care Inventory [33–35], and around –0.33 for the Diabetes Care Profile [36]. Of course, however, direct comparisons between those scales would be required to comparatively evaluate their properties.

Three independent reviews raised concerns against many available tools and encouraged the establishment of more appropriate ones [8–10]. Against this background, we consider the findings of this present study–supporting the DSMQ’s reliable and valid use to assess diabetes self-management in relation to glycaemic control–very promising.

There are some limitations of our study, primarily related to background and composition of the analysed convenience sample collected at a diabetes referral centre as well as the study’s cross-sectional nature. Patients are usually referred to this diabetes centre for problems of diabetes control, which is confirmed by the sample characteristics. The centre’s typical patient composition comprises up to 60% of patients with type 1 diabetes, and the majority of patients with type 2 diabetes is treated with insulin; both patient groups typically live with the condition for many years. Hence, the presented sample may not be generalisable for primary care settings. On the other hand, it is particularly this population in which the measurement of diabetes self-management to identify behavioural factors linked to poor glycaemic control may be desired. Since the data base for our study was cross-sectional, inferences of causal relationships between the assessed variables are not valid.

The strengths of the study lie in the standardised assessments (in-patient setting; psychometric assessment using standardised scales; blood sampling within few days apart from psychometric assessments; HbA_1c_ analysis in one central laboratory) as well as the sample composition comprising patients with both type 1 and type 2 diabetes, enabling generalisation for both major diabetes types.

In summary, this study supports the DSMQ as preferable tool when analysing behavioural problems related to reduced glycaemic control. The scale may be useful for clinical assessments of patients with suboptimal glycaemic outcomes or research on factors concerning associations between self-management behaviours and glycaemic control.

## Supporting Information

S1 Dataset SPSS 22.0.0 data file. (SAV)

S1 Table Diabetes Self-Management Questionnaire (DSMQ). (DOCX)

## References

[1] Inzucchi SE, Bergenstal RM, Buse JB, Diamant M, Ferrannini E, Nauck M, et al Management of hyperglycaemia in type 2 diabetes: a patient-centered approach. Position statement of the American Diabetes Association (ADA) and the European Association for the Study of Diabetes (EASD). Diabetologia. 2012 6;55(6):1577–96. 10.1007/s00125-012-2534-0 22526604

[2] Ahola AJ, Groop PH. Barriers to self-management of diabetes. Diabet Med. 2013 4;30(4):413–20. 10.1111/dme.12105 23278342

[3] Smith KJ, Rabasa-Lhoret R, Strychar I, Karelis AD, Clyde M, Levasseur J, et al Good vs. poor self-rated diabetes control: differences in cardiovascular risk and self-care activities. Exp Clin Endocrinol Diabetes. 2014 4;122(4):236–9. 10.1055/s-0034-1367005 24623501

[4] Fullerton B, Jeitler K, Seitz M, Horvath K, Berghold A, Siebenhofer A. Intensive glucose control versus conventional glucose control for type 1 diabetes mellitus. Cochrane Database Syst Rev. 2014 2 14;2:CD009122 10.1002/14651858.CD009122.pub2 24526393 PMC6486147

[5] Brown A, Reynolds LR, Bruemmer D. Intensive glycemic control and cardiovascular disease: an update. Nat Rev Cardiol. 2010 7;7(7):369–75. 20404853 10.1038/nrcardio.2010.35

[6] Stettler C, Allemann S, Jüni P, Cull CA, Holman RR, Egger M, et al Glycemic control and macrovascular disease in types 1 and 2 diabetes mellitus: Meta-analysis of randomized trials. Am Heart J. 2006 7;152(1):27–38. 16824829 10.1016/j.ahj.2005.09.015

[7] Toljamo M, Hentinen M. Adherence to self-care and glycemic control among people with insulin-dependent diabetes mellitus. J Adv Nurs. 2001 6;34(6):780–6. 11422548 10.1046/j.1365-2648.2001.01808.x

[8] Lu Y, Xu J, Zhao W, Han HR. Measuring Self-Care in Persons With Type 2 Diabetes: A Systematic Review. Eval Health Prof. 2015 6 30. pii: 0163278715588927. [Epub ahead of print] 10.1177/0163278715588927 PMC4792777 26130465

[9] Eigenmann CA, Colagiuri R, Skinner TC, Trevena L. Are current psychometric tools suitable for measuring outcomes of diabetes education? Diabet Med. 2009 4;26(4):425–36. 10.1111/j.1464-5491.2009.02697.x 19388974

[10] Caro-Bautista J, Martín-Santos FJ, Morales-Asencio JM. Systematic review of the psychometric properties and theoretical grounding of instruments evaluating self-care in people with type 2 diabetes mellitus. J Adv Nurs. 2014 6;70(6):1209–27. 10.1111/jan.12298 24237156

[11] Toobert DJ, Glasgow RE. Assessing diabetes self-management: the summary of diabetes self-care activities questionnaire In: Bradley C, editor. Handbook of Psychology and Diabetes: a guide to psychological measurement in diabetes research and practice. Switzerland: Harwood Academic; 1994 p. 351–75.

[12] Toobert DJ, Hampson SE, Glasgow RE. The summary of diabetes self-care activities measure: results from 7 studies and a revised scale. Diabetes Care. 2000 7;23(7):943–50. 10895844 10.2337/diacare.23.7.943

[13] Kamradt M, Bozorgmehr K, Krisam J, Freund T, Kiel M, Qreini M, et al Assessing self-management in patients with diabetes mellitus type 2 in Germany: validation of a German version of the Summary of Diabetes Self-Care Activities measure (SDSCA-G). Health Qual Life Outcomes. 2014 12 18;12:185 10.1186/s12955-014-0185-1 25519204 PMC4297436

[14] Primožič S, Tavčar R, Avbelj M, Dernovšek MZ, Oblak MR. Specific cognitive abilities are associated with diabetes self-management behavior among patients with type 2 diabetes. Diabetes Res Clin Pract. 2012 1;95(1):48–54. 10.1016/j.diabres.2011.09.004 21963107

[15] Amsberg S, Anderbro T, Wredling R, Lisspers J, Lins PE, Adamson U, Johansson UB. Experience from a behavioural medicine intervention among poorly controlled adult type 1 diabetes patients. Diabetes Res Clin Pract. 2009 4;84(1):76–83. 10.1016/j.diabres.2008.12.011 19181414

[16] Mayberry LS, Gonzalez JS, Wallston KA, Kripalani S, Osborn CY. The ARMS-D out performs the SDSCA, but both are reliable, valid, and predict glycemic control. Diabetes Res Clin Pract. 2013 11;102(2):96–104. 10.1016/j.diabres.2013.09.010 24209600 PMC3915929

[17] Lustman PJ, Clouse RE, Ciechanowski PS, Hirsch IB, Freedland KE. Depression-related hyperglycemia in type 1 diabetes: a mediational approach. Psychosom Med. 2005 Mar-Apr;67(2):195–9. 15784783 10.1097/01.psy.0000155670.88919.ad

[18] Egede LE, Osborn CY. Role of motivation in the relationship between depression, self-care, and glycemic control in adults with type 2 diabetes. Diabetes Educ. 2010 Mar-Apr;36(2):276–83. 10.1177/0145721710361389 20179250 PMC3085853

[19] Osborn CY, Bains SS, Egede LE. Health literacy, diabetes self-care, and glycemic control in adults with type 2 diabetes. Diabetes Technol Ther. 2010 11;12(11):913–9. 10.1089/dia.2010.0058 20879964 PMC3000637

[20] Heerman WJ, Wallston KA, Osborn CY, Bian A, Schlundt DG, Barto SD, Rothman RL. Food insecurity is associated with diabetes self-care behaviours and glycaemic control. Diabet Med. 2015 8 28 10.1111/dme.12896 [Epub ahead of print] PMC4769979 26314941

[21] Schmitt A, Gahr A, Hermanns N, Kulzer B, Huber J, Haak T. The Diabetes Self-Management Questionnaire (DSMQ): development and evaluation of an instrument to assess diabetes self-care activities associated with glycemic control. Health Qual Life Outcomes. 2013 8 13;11:138 10.1186/1477-7525-11-138 23937988 PMC3751743

[22] Schmitt A, Hermanns N, Kulzer B, Reimer A, Schall S, Haak T. The Diabetes Self-Management Questionnaire (DSMQ) can detect inadequate self-care behaviour and help identify patients at risk of a negative diabetes prognosis. 50th EASD Annual Meeting; 2014 Sep 15–19; Vienna, Austria. Diabetologia. 2014;57(Suppl.1):Abstract#1041.

[23] Nobis S, Lehr D, Ebert DD, Baumeister H, Snoek F, Riper H, Berking M. Efficacy of a web-based intervention with mobile phone support in treating depressive symptoms in adults with type 1 and type 2 diabetes: a randomized controlled trial. Diabetes Care. 2015 5;38(5):776–83. 10.2337/dc14-1728 25710923

[24] Brenk-Franz K, Strauss B, Tiesler F, Fleischhauer C, Ciechanowski P, Schneider N, et al The Influence of Adult Attachment on Patient Self-Management in Primary Care—The Need for a Personalized Approach and Patient-Centred Care. PLoS One. 2015 9 18;10(9):e0136723 10.1371/journal.pone.0136723 26381140 PMC4575213

[25] Nieminen E, Ferreira N. Diabetes acceptance and personal characteristics: Impact on health and behaviour outcomes in emerging adults with type 1 diabetes (T1D). Association for Contextual Behavioral Science (ACBS) Annual World Conference; 2015 Jul 15–18; Berlin, Germany. Available: https://contextualscience.org/wc13_posters

[26] Nieminen E. Diabetes acceptance and personal characteristics: Impact on health and behaviour outcomes in emerging adults with type 1 diabetes [thesis]. Edinburgh: The University of Edinburgh; 2015.

[27] Holcomb LS. Diabetes Type 2 Self-Management Education Program: Short Messaging from Patient Portal to Web-enabled Device [dissertation]. Valparaiso (IN): Valparaiso University; 2015.

[28] Hills SC. Impact of text messaging on diabetic foot self-care behaviors using a single-case design [dissertation]. Tyler (TX): University of Texas at Tyler; 2014.

[29] Navarro D. The Impact of Shift Work on Diabetes Self-management Activities: A Doctor of Nursing Practice Project [dissertation]. Las Vegas (NV): University of Nevada, Las Vegas; 2015.

[30] Nathan DM, Turgeon H, Regan S. Relationship between glycated haemoglobin levels and mean glucose levels over time. Diabetologia. 2007;50(11):2239–44. 17851648 10.1007/s00125-007-0803-0 PMC8752566

[31] Hu L, Bentler PM. Fit indices in covariance structure modeling: Sensitivity to underparameterized model misspecification. Psychol Methods. 1998;3(4):424–53.

[32] Templeton GF. A Two-Step Approach for Transforming Continuous Variables to Normal: Implications and Recommendations for IS Research. CAIS. 2011;28:4 Available: http://aisel.aisnet.org/cais/vol28/iss1/4/

[33] Khagram L, Martin CR, Davies MJ, Speight J. Psychometric validation of the Self-Care Inventory-Revised (SCI-R) in UK adults with type 2 diabetes using data from the AT.LANTUS Follow-on study. Health Qual Life Outcomes. 2013 2 26;11:24 10.1186/1477-7525-11-24 23443007 PMC3608221

[34] Lewin AB, LaGreca AM, Geffken GR, Williams LB, Duke DC, Storch EA, et al Validity and reliability of an adolescent and parent rating scale of type 1 diabetes adherence behaviors: the Self-Care Inventory (SCI). J Pediatr Psychol. 2009 10;34(9):999–1007. 10.1093/jpepsy/jsp032 19423660 PMC2782250

[35] Weinger K, Butler HA, Welch GW, La Greca AM. Measuring diabetes self-care: a psychometric analysis of the Self-Care Inventory-Revised with adults. Diabetes Care. 2005 6;28(6):1346–52. 15920050 10.2337/diacare.28.6.1346 PMC1615849

[36] Fitzgerald JT, Davis WK, Connell CM, Hess GE, Funnell MM, Hiss RG. Development and validation of the Diabetes Care Profile. Eval Health Prof. 1996 6;19(2):208–30. 10186911 10.1177/016327879601900205

