# Supplementary material for: Assessing Diabetes Self-Management with the Diabetes Self-Management Questionnaire (DSMQ) Can Help Analyse Behavioural Problems Related to Reduced Glycaemic Control
Source: PLoS One. 2016 Mar 3;11(3):e0150774. doi: 10.1371/journal.pone.0150774 (PMC4777391; doi:10.1371/journal.pone.0150774)
Supplement: S1 Table — (DOCX) [file pone.0150774.s002.docx]

**S1 Table**

**Diabetes Self-Management Questionnaire (DSMQ)**

| The following statements describe self-care activities related to your diabetes. Thinking about your self-care over the **last 8 weeks**, please specify the extent to which each statement applies to you. | | **applies to me very much** | **applies to me to a consider-able degree** | **applies to me to some degree** | **does not apply to me** |
| --- | --- | --- | --- | --- | --- |
| 1. | I check my blood sugar levels with care and attention.  *Blood sugar measurement is not required as a part of my treatment.* | 3 | 2 | 1 | 0 |
| 2. | The food I choose to eat makes it easy to achieve optimal blood sugar levels. | 3 | 2 | 1 | 0 |
| 3. | I keep all doctors’ appointments (appointments with health professionals) recommended for my diabetes treatment. | 3 | 2 | 1 | 0 |
| 4. | I take my diabetes medication (e. g. insulin, tablets) as prescribed.  *Diabetes medication / insulin is not required as a part of my treatment.* | 3 | 2 | 1 | 0 |
| 5. | Occasionally I eat lots of sweets or other foods rich in carbohydrates. | 3 | 2 | 1 | 0 |
| 6. | I record my blood sugar levels regularly (or analyse the value chart with my blood glucose meter).  *Blood sugar measurement is not required as a part of my treatment.* | 3 | 2 | 1 | 0 |
| 7. | I tend to avoid diabetes-related doctors’ appointments (appointments with health professionals). | 3 | 2 | 1 | 0 |
| 8. | I am regularly physically active to improve my diabetes treatment. | 3 | 2 | 1 | 0 |
| 9. | I strictly follow the dietary recommendations given by my doctor or diabetes specialist. | 3 | 2 | 1 | 0 |
| 10. | I do not check my blood sugar levels frequently enough to achieve good blood glucose control.  *Blood sugar measurement is not required as a part of my treatment.* | 3 | 2 | 1 | 0 |
| 11. | I avoid physical activity, although it could improve my diabetes. | 3 | 2 | 1 | 0 |
| 12. | I tend to forget to take or skip my diabetes medication (e. g. insulin, tablets).  *Diabetes medication / insulin is not required as a part of my treatment.* | 3 | 2 | 1 | 0 |
| 13. | Sometimes I have real ‘food binges’ (not triggered by hypoglycaemia). | 3 | 2 | 1 | 0 |
| 14. | Regarding my diabetes care, I should see my medical practitioner(s) more often. | 3 | 2 | 1 | 0 |
| 15. | I am less physically active than would be optimal for my diabetes. | 3 | 2 | 1 | 0 |
| 16. | My diabetes self-care is poor. | 3 | 2 | 1 | 0 |
